# Awareness of gestational diabetes mellitus and its associated factors among pregnant women in public hospitals in the East Gojjam Zone, Northwest Ethiopia: a cross-sectional study

**DOI:** 10.3389/fgwh.2025.1535344

**Published:** 2025-08-08

**Authors:** Getachew Tilaye Mihiret, Kumlachew Solomon Wondmu, Fekadu Baye, Mulunesh Minale, Mastewal Yechale, Misganaw Fikrie Melese, Aysheshim Belaineh Haimanot, Temesgen Getaneh

**Affiliations:** ^1^ Department of Midwifery, College of Medicine and Health Sciences, Debre Markos University, Debre-Markos, Ethiopia; ^2^ Department of Public Health, College of Medicine and Health Sciences, Debre Markos University, Debre-Markos, Ethiopia

**Keywords:** gestational diabetes mellitus, awareness, factors, pregnant women, Ethiopia

## Abstract

**Background:**

Gestational diabetes mellitus (GDM) is the most common metabolic complication during pregnancy and is associated with an increased risk of maternal and neonatal adverse outcomes. Despite it being the most prevalent complication and leading to poor pregnancy outcomes, there have been very few studies assessing awareness of GDM among pregnant women in Ethiopia. Therefore, this study aimed to determine the awareness of GDM and its associated factors among pregnant women in public hospitals in the East Gojjam Zone, Northwest Ethiopia.

**Methods:**

An institution-based cross-sectional study was conducted from June to August 2024. A systematic random sampling technique was utilized to select 423 participants. The data were collected using an interviewer-administered questionnaire. The collected data were analyzed using SPSS version 25. Binary logistic regression was fitted to assess the association between the explanatory variables and the outcome variable. Variables with a *p*-value less than 0.05, along with corresponding 95% confidence intervals, were used to declare statistical significance.

**Results:**

This study found that 27.0% (95% CI: 0.23–0.31) of the pregnant women were aware of GDM. The most common source of information about GDM was friends at 53.2%, followed by family and healthcare professionals. Factors such as partner involvement [adjusted odds ratio (AOR) = 0.58; 95% CI = (0.35–0.95)], family history of chronic medical conditions [AOR = 5.20; 95% CI = (2.40–11.25)], mistimed but wanted pregnancies [AOR = 3.36; 95% CI = (1.40–8.10)], and being Muslim [AOR = 2.89; 95% CI = (1.34–6.24)] were significantly associated with awareness of GDM.

**Conclusion:**

Only a small proportion of pregnant women were aware of GDM. Mistimed but desired pregnancies, partner involvement, and family history of chronic medical conditions were significantly associated with GDM awareness. In order to mitigate the growing burden of GDM, healthcare professionals need to do more to educate women about GDM during their prenatal care follow-ups.

## 1 Background

Gestational diabetes mellitus (GDM) is the presence of glucose intolerance during pregnancy that was not present before (1). Pregnancy often leads to insulin resistance, increasing the risk of developing diabetes (2). The main factors contributing to hyperglycemia in GDM are insulin resistance in peripheral tissues and inadequate insulin secretion by pancreatic beta-cells (3, 4).

Gestational diabetes mellitus is a common metabolic issue that occurs during pregnancy and is considered a significant public health concern with risks to the mother and child (5, 6). Globally, the prevalence of GDM varies from 1% to 28%, with an average prevalence of 15% (7). The International Diabetes Federation estimates that one out of six pregnancies is affected by diabetes, with GDM accounting for 86.4% of all cases of hyperglycemia during pregnancy (8). However, 87.6% of the burden and its consequences are found in low-and middle-income countries (LMICs), including in Africa, where obstetric and neonatal care is poor (9).

A review showed that the burden of GDM in the sub-Saharan African region was 14.3%, with the highest rates found in Central Africa at 20.4% and East Africa at 16.8%. A study conducted in Northwest Ethiopia showed that nearly 13% of women were diagnosed with GDM (10). This highlights the hidden challenges that GDM poses to the health of mothers and newborns in low- and middle-income countries such as Ethiopia.

Having gestational diabetes mellitus can lead to negative outcomes for the newborn, such as macrosomia, respiratory distress, premature birth, jaundice, hypoglycemia, stillbirth, and increased risk of neonatal intensive care unit admission (11–14). Additionally, neonatal GDM exposure raises the likelihood of hypertension, obesity, and type 2 diabetes mellitus (T2DM) later in life (15, 16). Gestational diabetes is also associated with a higher risk of adverse pregnancy outcomes, such as cesarean delivery, pregnancy-induced hypertension, premature rupture of membranes, antepartum hemorrhage, and postpartum hemorrhage (5, 14, 15, 17). Generally, GDM is a disease with high health and economic costs, but can be prevented or mitigated through proper antenatal screening, prompt diagnosis, early initiation of treatment, and ongoing monitoring, in which awareness is the baseline (13).

In the developed world, pregnant women are routinely screened for GDM. Despite sharing the highest burden in terms of prevalence and poor pregnancy outcomes, women are rarely screened for GDM in low- and middle-income countries (18). This can be due to economic issues and health system policies that have not implemented the recommendation of universal screening. However, the low screening rates are primarily due to a lack of awareness among pregnant women (19).

Awareness of GDM among pregnant women could be a key strategy in the primary prevention of the disease. This is because awareness of the condition among pregnant women leads to the adoption of healthy lifestyles and better health-seeking behaviors, including early screening, diagnosis, and management of the disease, all of which improve outcomes (20, 21).

A review of evidence confirmed that improving women's awareness of GDM is crucial for prevention, early management, and improving the long-term outcomes of the condition (22). In LMICs, very few studies have assessed the awareness of GDM among pregnant women (23), including in Ethiopia. Most of the available research conducted in Ethiopia has shown the burden of GDM, its associated factors, and its adverse outcomes.

Determining the existing GDM awareness gap among pregnant women is necessary to tackle the problem, plan appropriate strategies, and improve maternal and child health. Therefore, this study aimed to assess GDM awareness and its associated factors in Northwest Ethiopia.

## 2 Methods

### 2.1 Study area and period

This study was conducted from June to August 2024 in public hospitals in East Gojjam. The zone is found in the Amhara Region of Ethiopia, bordered to the south by the Oromia Region, to the west by West Gojjam, to the north by Debub Gondar, and to the east by Debub Wollo. The zone had an estimated population of 2,719,118 in 2020 (24). Based on the 2019 East Gojjam Zone Administration Office report, the zone had an estimated 91,634 women of reproductive age. This zone has 10 public hospitals, including one general hospital, eight primary hospitals, and one comprehensive specialized hospital, as well as 104 health centers and 406 health posts (25).

### 2.2 Study design

An institution-based cross-sectional study design was utilized.

### 2.3 Population

#### 2.3.1 Source population

All pregnant women who had antenatal care (ANC) follow-ups at the public hospitals in the East Gojjam Zone.

#### 2.3.2 Study population

All eligible pregnant women who had antenatal care follow-ups at the public hospitals in the East Gojjam Zone during the actual data collection period

### 2.4 Eligibility criteria

All pregnant women who had antenatal care follow-ups and were available at the time of data collection were included in this study, while pregnant women with pre-existing type-1 or type-2 diabetes and those who were seriously sick or unable to communicate during data collection were excluded.

### 2.5 Sample size determination

The assumption of the single population proportion formula was utilized to compute the sample size.

$$n=\;\frac{(Z\alpha /2)\;2p\;(1-p)\;}{\;d2}$$

where *n* = the desired sample size, Z*α*/2 = the critical value corresponding to a 95% confidence interval (Z*α*/2 = 1.96), d = the margin of error (5%), and p = the estimated population proportion, which was 48% from the study conducted in Oromia, Ethiopia (26).

$N=\frac{(1.96)2\times \;0.48\times (1-0.0.48)\;}{\;\;(0.05)2}=383.5∼384$.

After considering a 10% non-response rate, the final sample size (Nf) = ni × (1/1-non-response rate); thus, Nf = 384 × (1/1–0.1) = 427.

### 2.6 Sampling procedure and method

A sample of five public hospitals was selected using a simple random sampling technique from the 10 public hospitals located in the East Gojjam Zone. The antenatal care registration book of each hospital was used to proportionally allocate the calculated sample size and determine the sampling fraction (k) (calculated using the population size divided by the sample size). The first mother was chosen using a simple random sampling technique among mothers who had an antenatal care follow-up on the day of data collection. Then, a systematic random sampling technique was used to select participants until the required sample size was achieved.

### 2.7 Study variables

#### 2.7.1 Dependent variable

⮚ Awareness of GDM

#### 2.7.2 Independent variables

⮚ Sociodemographic variables
⮚ Obstetric-related variables
⮚ Lifestyle and medical disease-related variables

### 2.8 Data collection tool and procedure

The data were collected using a face-to-face interviewer-administered questionnaire adapted from previously published articles (26, 27). The tool included the patient's background information and obstetric and medical history, as well as 15 questions assessing awareness of GDM, including its risk factors, diagnosis, treatment, and complications. The questions on risk factors assessed the patient’s awareness of the risk of GDM in patients with obesity prior to pregnancy, excessive weight gain during the present pregnancy, history of diabetes mellitus during previous pregnancies, and family history of diabetes.

Awareness of the course of GDM and its consequences to the unborn baby and mother was assessed by questions on whether GDM usually disappeared after delivery and whether women with GDM and children born to these women were at an increased risk for future T2DM and obesity.

To assess the patient’s awareness of the screening and diagnosis of GDM, questions on the type of test used and the timing of the test during pregnancy were asked. The options for the type of test used were urine test, blood test, blood test after a glucose load, and I do n't know. For the timing of the test, the options given were 12–16 weeks (3–4 months), 24–28 weeks (6–7 months), during delivery, and I do n't know. The answer 24–28 weeks (6–7 months) was considered the correct answer.

The patient’s awareness of the treatment for GDM was assessed using a question with the following options: diet and exercise, oral antidiabetic drugs, insulin injections, and I do n't know. Diet and exercise, insulin injections, and oral antidiabetic drugs were considered the correct response. Each correct response was given a score of 1 and each woman was scored out of a total of 15.

The tool was prepared in English and then translated into Amharic (the local language). It was then translated back into English to check its consistency. The face and content validity of the Amharic version questionnaires were assessed by four experts to control the threat to the validity of the data from the proposed instrument. In addition, the tool was pretested with 22 pregnant women who met the study criteria and was revised by experienced academic researchers. A well-structured tool, consisting of a chart review and interviewer-administered questions, was utilized to collect the data. The tool included sociodemographic information, obstetric and medical-related data, lifestyle-related data, and information on awareness of GDM. Five BSc midwives and five MSc professionals were recruited as data collectors and supervisors, respectively.

### 2.9 Assurance of data quality

A 2-day training session was provided to both data collectors and supervisors by the principal investigator about the objective of the study, the data collection tool, procedure, and how to fill out the questionnaire. The tool was pretested at Fenote Selam public hospital on 5% of the sample size to ensure the consistency and completeness of the questionnaire. Data collectors were supervised throughout the course of the data collection period. Then, the overall process was coordinated and controlled by the principal investigator. The principal investigator, supervisors, and data collectors had a discussion meeting after data collection to ensure completeness. Codes were given to the questionnaires during data collection. Furthermore, the collected data were entered into EpiData version 4.2 to minimize data entry errors and kept in the form of a file in a secure place. The results of the study were used only for the study's purpose.

### 2.10 Data analysis

The collected data were entered into EpiData version 4.2. It was then exported to SPSS version 25 for analysis. Descriptive statistics, such as frequency and summary statistics, were used to describe the characteristics of the study participants. A binary logistic regression model was used to determine the factors associated with the outcome variable. The model's fitness was assessed using the Hosmer–Lemeshow goodness-of-fit test for the adjusted model [chi-square (*χ*²) = 8.93, *p* = 0.35], which indicated a good fit. Multicollinearity among the independent variables was evaluated using variance inflation factors (VIFs), and no significant multicollinearity was detected. In the bivariable logistic regression, all explanatory variables with a *p*-value of 0.25 or less were considered for the multivariable logistic regression analysis. An adjusted odds ratio (AOR) with its corresponding 95% confidence intervals was used to indicate the association between the dependent and independent variables, and a *p*-value less than 0.05 indicated statistical significance.

### 2.11 Ethical clearance

Ethical clearance was obtained from the Institutional Ethical Review Board of the College of Medicine and Health Sciences, Debre Markos University (Approval No.: CMHS/R/C/Ser/D/315/01/16). Responsible officials and managers at each hospital were informed and permission was obtained. The participants (legal guardian/next of kin) provided written informed consent to participate in this study, and were informed that they had the right to withdraw from the study at any time.

### 2.12 Operational definitions of variables

The awareness score was determined based on the participants’ correct answers, with 1 point given for each correct answer and 0 for incorrect responses. It was measured using 15 awareness questions and categorized as “good awareness of GDM” (>9 out of 15), “fair awareness of GDM” (6–9 out of 15), and “poor awareness of GDM” (0–5 out of 15). Finally, pregnant women with good or fair knowledge were considered to be aware of GDM, and those with poor knowledge were considered to be unaware (21, 23).

## 3 Results

### 3.1 Demographic characteristics of participants

In total, 423 (99.1%) respondents participated in this study. The mean maternal age of the participants was 26.64 (SD 4.96) years, of which 63.6% were ≥25 years. Almost all of the respondents (421, 99.5%) were married. Approximately 30% of the study participants had attended college or higher education, while 56 (13.2%) had not received any formal education. Over half (51.8%) of the study participants were housewives (Table 1).

**Table 1:** Demographic characteristics of the study participants in the East Gojjam Zone public hospitals, Northwest Ethiopia, in 2024 (*n* = 423).

Variable	Category	Frequency	Percentage (%)
Age	15–24	154	36.4
	25–34	227	53.7
	≥35	42	9.9
Residence	Urban	334	79.0
	Rural	89	21.0
Religion	Orthodox	381	90.1
	Muslim	42	9.9
Maternal education	No formal education	56	13.2
	Primary school	122	28.8
	Secondary school	117	27.7
	College and above	128	30.3
Maternal occupation	Housewife	219	51.8
	Employed	70	16.5
	Merchant	58	18.0
	Farmer	111	13.7
Husband education	No formal education	52	12.3
	Primary school	90	21.3
	Secondary school	103	24.3
	College and above	176	41.6
Husband occupation	Daily labor	13	3.1
	Farmer	75	17.7
	Employed	208	50.2
	Merchant	113	26.7
	Others^a^	12	2.9
Monthly income (in ETB)	<5,000	96	22.7
	≥5,000	327	77.3
Family size	1–2	125	29.6
	3–4	195	46.1
	≥5	103	24.3

^a^ Priest and car driver.

### 3.2 Maternal obstetrical characteristics

More than three-fifths (63.1%) of the women were multigravida; 393 (92.9%) of the women who participated had experienced a planned and wanted pregnancy. Approximately 12% (52, 12.3%) of the participants had a history of a bad obstetric complication, with abortions accounting for 71.2% of these cases, followed by early neonatal death and stillbirth in 17.3% and 11.5%, respectively. Approximately half (214, 50.5%) of the study participants were in their second trimester of pregnancy (Table 2).

**Table 2:** Obstetrical characteristics of the pregnant women in the East Gojjam Zone public hospitals, Northwest Ethiopia, in 2024 (*n* = 423).

Variable	Category	Frequency	Percentage (%)
Parity	Nulliparous	184	43.5
	1–2	195	46.1
	≥3	44	10.4
No. of pregnancy	Singleton	419	99.1
	Twin	4	0.9
Gestational age at which ANC started	First trimester	188	44.4
	Second trimester	214	50.6
	Third trimester	21	5.0
No. of ANC appointments	1–2	135	31.9
	3–4	81	19.2
	4–5	119	28.1
	6–8	88	20.8
Did you receive tetanus injection?	Yes	277	65.5
	No	146	34.5
Iron and folic acid utilization	Yes	398	94.1
	No	25	5.9
Duration of iron utilization	For 1 month	87	21.9
	For 2 months	101	25.3
	For 3 months or more	210	52.8
Partner involvement	Yes	215	50.8
	No	208	49.2

### 3.3 Lifestyle and medical history-related factors

Of the study participants, 8 (1.9%) had chronic medical disease, among which HIV accounted for 50% of these cases, followed by hepatitis and psychosis in the remaining 50%. All HIV-positive participants received antiretroviral therapy. Approximately 10% of the participants had a family history of chronic medical disease, with diabetes mellitus and hypertension being the most commonly noted conditions (Table 3).

**Table 3:** Lifestyle and medical history-related characteristics of the study participants.

Variable	Category	Frequency	Percentage (%)
High blood pressure	Yes	14	3.3
	No	409	96.7
Cardiac disease	Yes	1	0.2
	No	422	99.8
Renal disease	Yes	16	3.8
	No	407	96.2
Family history of diabetes mellitus	Yes	34	8.0
	No	389	92.0

### 3.4 Awareness of gestational diabetes mellitus

**Figure 1:**
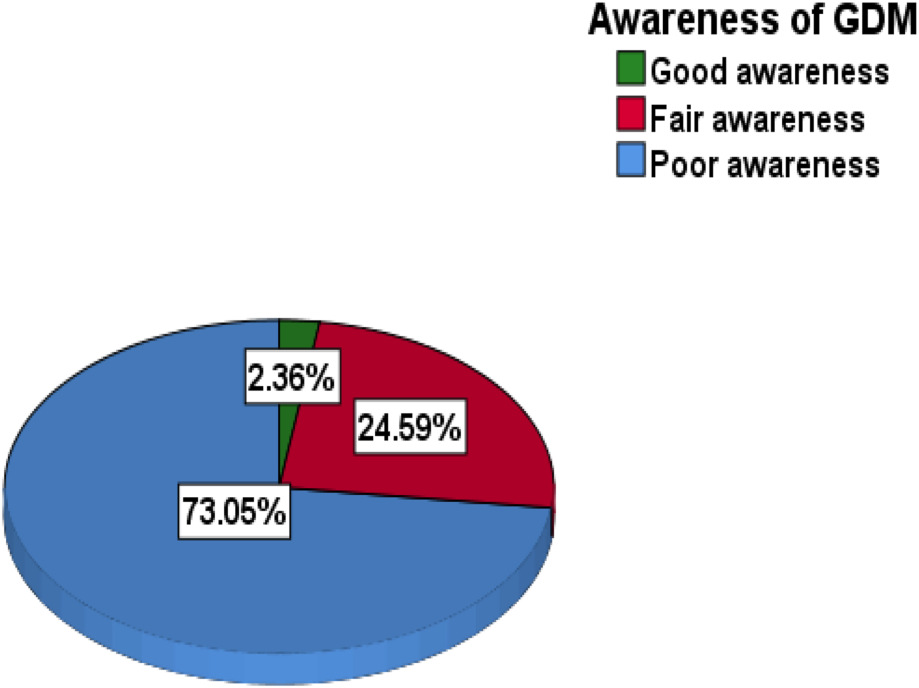
Levels of gestational diabetes mellitus awareness among pregnant women at public health institutions in the East Gojjam Zone public hospitals, Northwest Ethiopia, in 2024.

Participants were grouped into three categories based on their scores, with “good awareness of GDM” defined as a score of >9 out of 15, “fair awareness” as a score of 6–9 out of 15, and “poor awareness” as a score of 0–5 out of 15. Ultimately, women with good or fair awareness were considered to be aware of GDM, while those with poor knowledge were deemed to be unaware. Accordingly, the research revealed that only 27.0% (95% CI: 0.23–0.31) of the pregnant women were aware of GDM (Figure 1).

### 3.5 Source of information about GDM

**Figure 2:**
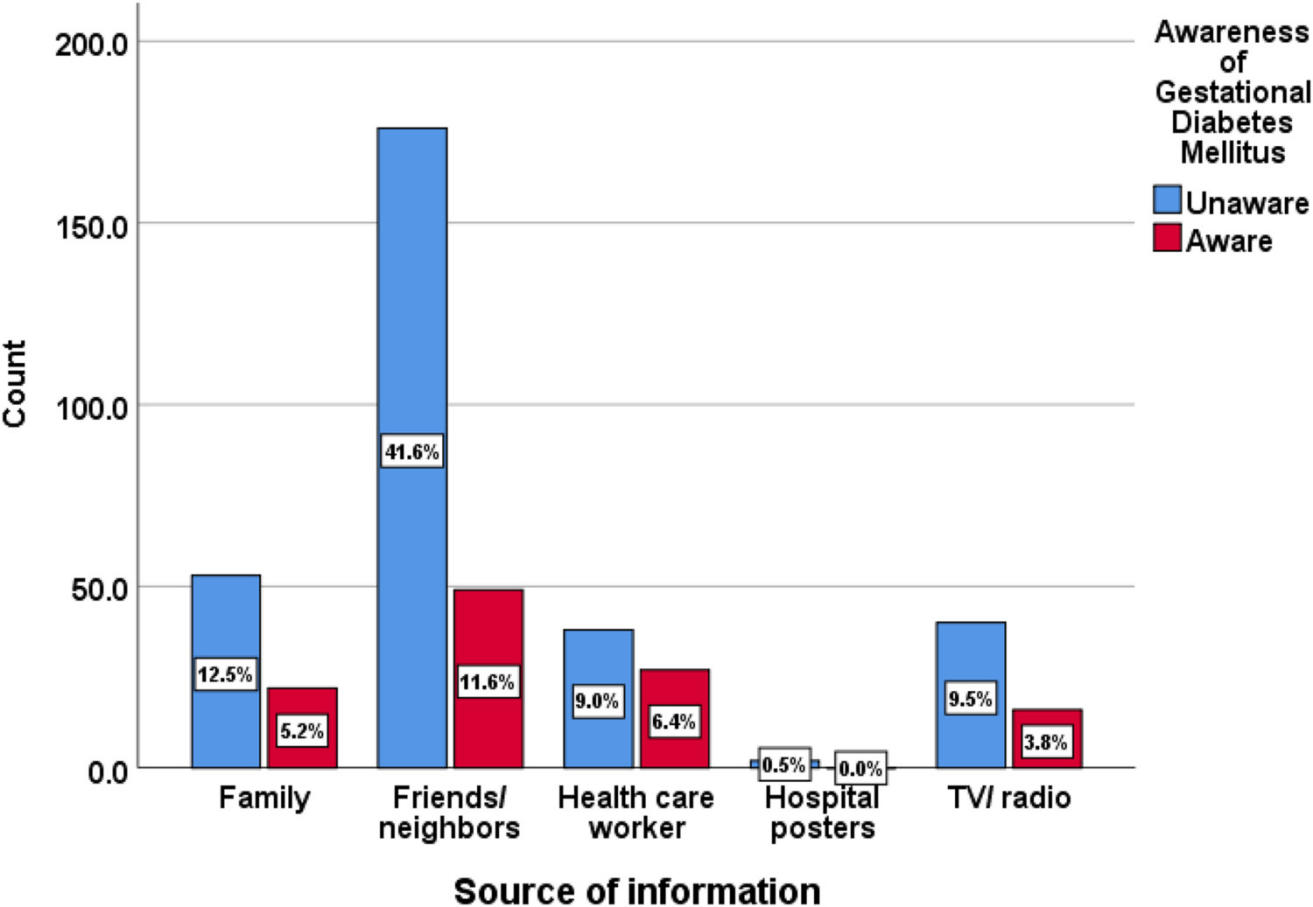
Sources of information about gestational diabetes mellitus.

Various sources of information about GDM were identified, with friends being the most common at 53.2%, followed by family members at 17.7% and healthcare professionals at 15.4% (Figure 2).

### 3.6 Factors associated with GDM awareness

In the bivariable logistic regression model, we considered various factors for further analysis, including maternal age, residence, religion, occupation, income, parity, receipt of tetanus toxoid injection and iron supplementation, pregnancy status, family history of medical disease, obstetrical history, past medical conditions, and partner involvement (all with *p* < 0.25).

In the final multivariable logistic regression model, several factors emerged as significantly associated with awareness of GDM. Women who were Muslim were 2.9 times more likely to have good awareness compared to Orthodox women (CI 1.34, 6.24; *p* = 0.007). Partner involvement also played a role; women who did not have an involved partner were less likely to have good awareness compared to those with involved partners (AOR: 0.58; CI 0.35, 0.95; *p* = 0.03). A family history of chronic medical disease (diabetes, hypertension, or both) increased the likelihood of good GDM awareness by 5.2 times (CI 2.40, 11.25; *p* < 0.001). Additionally, women with mistimed but wanted pregnancies were 3.4 times more likely to have good GDM awareness compared to those with planned and wanted pregnancies (CI 1.40, 8.10; *p* = 0.007) (Table 4).

**Table 4:** Factors associated with awareness of GDM among pregnant women (*n* = 423).

Variable	Category	Awareness of GDM		Odds ratio (95% CI)	
		Aware	Unaware	COR	AOR
Age	15–24	27	127	1	1
	25–34	72	155	2.19 (1.32, 3.61)	1.70 (0.89, 3.28)
	≥35	15	27	2.61 (1.23, 5.56)	2.92 (0.89, 9.51)
Residence	Urban	103	231	1	1
	Rural	11	78	0.32 (0.16, 0.62)	0.99 (0.35, 2.86)
Religion	Orthodox	95	286	1	1
	Muslim	19	23	2.49 (1.30, 4.77)	2.89 (1.34, 6.24)**
Maternal occupation	Employed	5	53	1	1
	Housewife	58	161	0.57 (0.33, 1.01)	0.79 (0.40, 1.58)
	Merchant	24	52	0.74 (0.37, 1.45)	0.87 (0.40, 1.88)
	Farmer	27	43	0.15 (0.05, 0.42)	0.22 (0.05, 1.00)
Companied monthly income	<5,000 ETB	17	79	1	1
	≥5,000 ETB	97	230	1.96 (1.10, 3.48)	1.36 (0.68, 2.73)
Parity	Nulliparous	40	144	0.40 (0.20, 0.80)	1.12 (0.38, 3.80)
	1–2	56	139	0.58 (0.29, 1.14)	0.99 (0.35, 2.79)
	≥3	18	26	1	1
Having a chronic medical disease	Yes	28	14	6.86 (3.46,13.61)	5.20 (2.40,11.25) **
	No	86	295	1	1
Bad obstetrical history	Yes	9	43	1.89 (0.89, 4.00)	0.42 (0.18, 1.0)
	No	105	266	1	1
Pregnancy status	Intended pregnancy	101	292	1	1
	Mistimed, yet wanted	13	17	2.21 (1.04, 4.71)	3.36 (1.40, 8.10)**
Receiving a tetanus toxoid injection	Yes	88	189	2.15 (1.31, 3.52)	1.71 (0.94, 3.13)
	No	26	120	1	1
Partner involvement	Yes	66	142	1.62 (1.05, 2.50)	0.58 (0.35, 0.95)*
	No	48	167	1	

* *p* < 0.05; ** *p* < 0.01; 1 = Reference; COR, crude odds ratio; Hosmer–Lemeshow test for the adjusted model: chi-square = 8.93, *p* = 0.35.

## 4 Discussion

This study evaluated awareness of GDM and its associated factors among pregnant women in public hospitals in the East Gojjam Zone, Northwest Ethiopia. In this study, only 27% of the participants were aware of GDM. This finding is consistent with previous studies conducted in India (22%) (20), Uganda (31%) (23), Saudi Arabia (28), Nigeria (28.8%) (18), and Kenya (29.0%) (29). This implies that awareness of GDM among women is relatively comparable across different countries, despite variations in socioeconomic status.

However, the finding of this study was not comparable with other studies conducted in Ethiopia (48%) (26), Bangladesh (89.4%) (30), Nigeria (86.9%) (31), Egypt (69.6%) (27), and India, which reported awareness levels of 41.7% (22) and 74.4% (21). These variations could be attributed to differences in access to information. The studies conducted in Nigeria (86.9%) (31) and Egypt (69.6%) (27) reported that healthcare professionals were the primary source of information, while in this study, the most frequently cited sources of information about GDM were friends, followed by family and mass media. These findings emphasize the limited role of healthcare providers in Ethiopia regarding disseminating information about GDM. This highlights the need for improved communication strategies within the healthcare system to raise awareness among pregnant women and mitigate the growing burden of GDM.

The current study found that having a family history of chronic medical conditions was associated with increased awareness of GDM. This finding is comparable with previous studies conducted in Central Ethiopia and Bangladesh (30), which reported that a family history of diabetes was significantly linked to a higher awareness of GDM. This implies that women with affected relatives may be more familiar with the condition and its characteristics, leading to a better understanding of GDM (26).

This study also found a significant religious difference in awareness of GDM, with Muslim women being 2.9 times more likely to be aware of GDM compared to Orthodox Christian women. This may be due to the majority of Muslim participants in this study residing in urban areas, where access to health education and other social facilities that promote GDM awareness is readily available. This finding was also supported by prior studies conducted in India (20) and Kenya (32); they found similar findings regarding religious differences in GDM awareness among pregnant women.

Moreover, the study found that partner involvement in antenatal care follow-up plays a pivotal role in boosting awareness of GDM. Pregnant women without partner involvement were 42% less likely to be aware of GDM compared to those whose partners were involved. Previous studies have also supported this finding (33, 34). This indicates the importance of considering partner-based strategies in antenatal care follow-up, which can improve awareness and self-management of GDM.

Women who had mistimed but desired pregnancies were 3.4 times more likely to be aware of GDM compared to those with intended pregnancies. This is supported by previous studies (35, 36). A possible reason for this finding may be that a woman who has mistimed but still desired pregnancy may feel more pressure to have a healthy pregnancy. This could lead them to be more proactive in seeking out prenatal care, including health information on potential risks such as GDM.

## 5 Conclusion

Only a small proportion of the pregnant women in this study were aware of GDM. The most common sources of information about GDM were friends and family. Mistimed but desired pregnancies, partner involvement, and a family history of chronic medical conditions were significantly associated with GDM awareness. In order to mitigate the growing burden of GDM, healthcare professionals need to do more to educate women about GDM during their prenatal care follow-ups.

## Data Availability

The raw data supporting the conclusions of this article will be made available by the authors, without undue reservation.
